# Chunking, boosting, or offloading? Using serial position to investigate long-term memory's enhancement of verbal working memory performance

**DOI:** 10.3758/s13414-022-02625-w

**Published:** 2022-12-01

**Authors:** Lea M. Bartsch, Peter Shepherdson

**Affiliations:** 1 Department of Psychology, University of Zurich, Zurich, Switzerland; 2 Department of Psychology, University of Akureyri, Akureyri, Iceland

**Keywords:** Working memory, Long-term memory, Position effects

## Abstract

Individuals can use information stored in episodic long-term memory (LTM) to optimize performance in a working memory (WM) task, and the WM system negotiates the exchange of information between WM and LTM depending on the current memory load. In this study, we assessed the ability of different accounts of interactions between LTM and WM to explain these findings, by investigating whether the position of pre-learnt information within a memory list encoded into WM affects the benefit it provides to immediate memory. In two experiments we varied the input position of previously learned word-word pairs within a set of four to-be-remembered pairs. We replicated previous findings of superior performance when these LTM pairs were included in the WM task and show that the position in the list in which these LTM pairs were included not seem to matter. These results are most consistent with the idea that having access to information in LTM reduces or removes the need to rely on WM for its storage, implying that people “offload” information in conditions containing LTM pairs.

**Supplementary Information:**

The online version contains supplementary material available at 10.3758/s13414-022-02625-w.

## Introduction

Interactions between working memory (WM) and long-term memory (LTM) have been a feature of memory theories dating back many decades (Atkinson & Shiffrin, 1968). WM and LTM show considerable structural and functional overlap (Eriksson et al., 2015; Lewis-Peacock & Postle, 2008; Ranganath et al., 2005), and several current memory models conceptualize WM as a subset of LTM representations that are in a temporarily heightened state of accessibility (Cowan, 1995; McElree, 1998; Oberauer, 2002; but see Baddeley, 2012; Barrouillet & Camos, 2012; Unsworth & Engle, 2007b). For instance, the three-embedded components model postulates ﻿that WM comprises three functionally distinct yet overlapping parts: (1) The activated part of LTM, (2) the region of direct access, and (3) the single-item focus of attention (Oberauer, 2002; Oberauer & Hein, 2012). The region of direct access is characterized by holding a limited number of chunks available to be used in ongoing cognitive processes, reflecting what is commonly referred to as the limited capacity of WM (see, e.g., Cowan, 2001).

### Interaction of working (WM) and long-term memory (LTM)

Despite the theoretical connection of LTM and prior knowledge with WM, the specific manner in which prior knowledge is used in WM and thereby how it affects performance in immediate memory tasks^1^ remains unclear. Importantly, within conceptualizations of LTM, a distinction is often made between *semantic* and *episodic* LTM (see Rubin, 2022, for a recent review) and the empirical evidence for contributions of both types of LTM to performance in immediate memory tasks varies in scope. Episodic LTM refers to memory for the personal experience of past events. This can include, for example, experiences that individuals have been exposed to in a laboratory setting, including memory for lists of words or pictures presented to them. Semantic LTM refers to our knowledge of facts, including the meaning of words, and representations of well-known concepts.

Knowledge from *semantic* LTM is assumed to contribute to performance in most tests of WM, as shown in effects like the sentence superiority benefit, the chunking benefit, and the lexicality effect in WM (Baddeley et al., 2009; Engle et al., 1992; Hulme et al., 1991; Jefferies et al., 2006; Romani et al., 2008; Thalmann et al., 2019). Additionally, substantial evidence shows superior performance on tasks requiring WM when materials are familiar (e.g., Charness, 1991; Chase & Simon, 1973; Ericsson & Kintsch, 1995; Xie & Zhang, 2017). Taken together, there is thus consensus that *semantic* LTM contributes generally to performance in immediate memory tests that are commonly used to assess WM (Kowialiewski & Majerus, 2018; Poirier & Saint-Aubin, 1995; Walker & Hulme, 1999).

The evidence regarding whether and how *episodic* LTM provides an additional contribution is less extensive. Prior work has shown that measures of WM capacity are highly correlated with the ability to remember over the long term (Unsworth, 2010; Wilhelm et al., 2013), and there have been numerous suggestions that items in immediate memory tasks can be retrieved from LTM using temporal-contextual cues (Raaijmakers & Shiffrin, 1980; Shiffrin & Atkinson, 1969; Unsworth & Engle, 2006a, 2006b, 2007a, 2007b). Responses in these tasks thus appear to result from a combination of representations maintained in the short-term buffer, and information retrieved from episodic LTM. Indeed, Beukers et al. (2021) have recently argued that contributions from “activity-silent” WM (i.e., representations not currently subject to active maintenance) may simply reflect information retrieved from episodic memory.

Some researchers have also shown superior performance in immediate memory tasks with materials designed to allow reliance on episodic memory. For instance, Chen and Cowan (2005, 2009) showed that participants had better recall for lists containing pairs of words that had been learned in an earlier phase of the experiment, compared to lists with novel pairings of words. Similarly, we (Bartsch & Shepherdson, 2022) previously investigated whether the presence and use of episodic LTM representations frees capacity for maintaining additional information in WM, showing that immediate memory task performance was enhanced when LTM representations were available and reliable. In combination, these findings indicate that immediate recall of verbal lists benefits from prior episodic LTM traces.

### Why should the position of LTM information matter?

Research on the effects of *semantic* LTM in the form of chunks in WM (e.g. “PDF” in a list of letters) has provided evidence that only lists with chunks presented in the beginning of a list freed WM capacity – allowing more storage for new information (Thalmann et al., 2019). This benefit was assumed to result from the recoding of individual items into a chunk, and their subsequent removal from WM, freeing capacity for items that followed (Thalmann et al., 2019). When no further items followed – that is, when the chunkable items were presented at the end of a trial – the benefit was thus minimal.

Here, we aim to investigate whether this position-related boundary condition also holds for the effect of *episodic* LTM on immediate memory performance, and thus elucidate whether similar mechanisms underlie benefits to WM from both semantic and episodic LTM. In our previous study (Bartsch & Shepherdson, 2022), pairs of words that were previously learned (“LTM-available” pairs) were included at random positions within a list of pairs in the WM task. One of the words from a pair was then used as a cue to remember the other. The pre-learnt bindings facilitated performance, but the unsystematic presentation of LTM-available and new pairs within lists complicated analysis of positional effects. The experiments reported here address this issue.

We can identify three different accounts of how representations retained in episodic LTM might enhance immediate memory performance. First, according to the *chunking account*, this benefit may come about in a manner analogous to that suggested in Thalmann et al.’s (2019) research on semantic LTM. Specifically, the existence of representations in LTM that match materials presented in an immediate memory task may obviate the need to retain that information directly in WM. Instead, a reference to the relevant chunk in LTM (for our purposes, a word pair) can take its place, and – assuming this reference places less strain on the limited capacity of WM than the information it replaces (i.e., the individual words and their binding) – the load placed on WM is thereby reduced.

Second, according to the *trace strength* account, building on the idea that retrieval from episodic LTM plays an integral role in WM tasks (e.g., Beukers et al., 2021; Unsworth & Engle, 2007b), having a pre-existing representation in LTM that matches content presented in such a task may facilitate this process. For instance, a stronger trace in episodic memory for material already stored in LTM could increase the probability of successful retrieval of these LTM items. Additionally, a strong trace in episodic LTM could reduce confusion with other information in WM, by enhancing rejection of mis-bound words through retrieval of correct pairings.

These two accounts were those we initially proposed prior to commencing the first experiment we report here. However, the results of that experiment prompted us to consider an additional “offloading” account, in which LTM-available stimuli are not encoded into WM at all, freeing capacity for the retention of LTM-unavailable stimuli. This would result in a benefit from the presence of LTM-available stimuli that is independent of their serial position and would lead to generally superior performance for new items.

All three of these accounts make similar predictions regarding overall effects on immediate memory performance: Generally, people’s memory should be superior when they are presented with memory sets that contain at least some pre-learnt materials. However, they differ in their predictions concerning the importance of position in the impact of LTM on performance. As mentioned earlier, according to the chunking account, items (e.g., words, letters) are first encoded to WM individually, before being replaced by a single chunk when this is possible (e.g., where letters make a familiar acronym, or a pair of words is already stored in LTM). This frees capacity for subsequent encoding of additional items. As a result, benefits of LTM should be greatest when capacity is freed *before* other materials are presented and must be remembered (i.e., at the beginning of a list) and least when capacity is freed *after* the presentation of other materials has concluded (i.e., at the end of a list). By contrast, according to the trace strength and the offloading accounts, there is no reason to assume that the impact of having an existing episodic LTM trace will differ with the position in a list in which that information is presented.

## The present study

In the present study we investigated whether the position of stimuli available in LTM within a list of new stimuli affects their impact on immediate memory performance in a WM task. In the first phase of each experiment, we presented participants with word pairs for them to encode into LTM (*LTM learning phase*). Subsequently, they completed trials of a WM task, also involving word pairs. The pairs presented in each WM trial consisted of varying numbers of new pairs (LTM unavailable) and the previously learned LTM pairs. Crucially, within a four-pair list, the LTM pairs were presented at the beginning (positions 1 and 2), the middle (positions 2 and 3), or the end (positions 3 and 4). Our main goal was to test the hypothesis that the presence of an LTM pair within a WM task frees resources to encode subsequent items more robustly – similar to the chunking benefit derived from semantic LTM (Thalmann et al., 2019). If so, this should result in a stronger benefit of the presence of LTM pairs when they are presented at the beginning of the list, relative to other positions.

In the first experiment, we examined whether and how input position moderated the effect the LTM pairs on participants’ WM test performance, which we thought would help distinguish our hypothesised chunking account from an alternative trace strength account. Contrary to our hypothesis, the effects of input position seemed limited, a result more consistent with the trace strength than the chunking account. However, generally superior performance at later positions in all conditions complicated this conclusion, leading us to posit a unique contribution from the focus of attention (FoA), and to propose a third account in which encoding of chunks’ constituent parts into WM is not necessary, and information is “offloaded” to LTM entirely.

In the second experiment, we replicated the findings of the first experiment and extended the design by inclusion of a distractor task in the retention interval. In doing so we aimed to assess the idea that memory for LTM pairs – when included in the WM task – is offloaded to long-term memory without requiring initial encoding into WM. This would result in freeing of capacity within WM that does not depend on the LTM pairs’ serial positions, and leads to superior performance for new items in all cases. The trace strength account would produce a similar outcome, but the two accounts differ in their predictions regarding the effects of distractors on memory when LTM-available pairs are tested, with only the offloading account predicting protection of these stimuli from distractor effects. Further, we wanted to test the role of the FoA in performance: by including a distractor task, the FoA would be filled with the distractor stimuli, rather than the last presented item(s) of the list. As predicted, the inclusion of a distractor task during the retention interval largely reduced the enhancement of memory for new pairs (though with some important exceptions), which the ability to offload LTM pairs would otherwise have provided: Essentially, the freed capacity obtained by offloading the LTM pairs is counteracted by interference from the distractor task, but only for novel stimuli.

## Experiment 1

### Method

#### Participants

We collected data from 99 participants online via Prolific (M_age_ = 25.92 years, 62 female). We excluded 11 participants as their response accuracy in the final LTM test was below or at chance level. Therefore, the final sample included 88 participants. All participants’ first language was English. Participants of this and Experiment 2 gave informed consent prior to the study and were debriefed at the end. We chose the sample size for this and the second experiments because it was sufficient to detect the effects of interest in a previous within-subject design, which was also run online (see Bartsch & Shepherdson, 2022, Exp. 3). The use of Bayesian statistics means that the sample size could have been increased in case of ambiguous evidence (Rouder, 2014). This was necessary for Experiment 2.

#### Materials and procedure

**Fig. 1:**
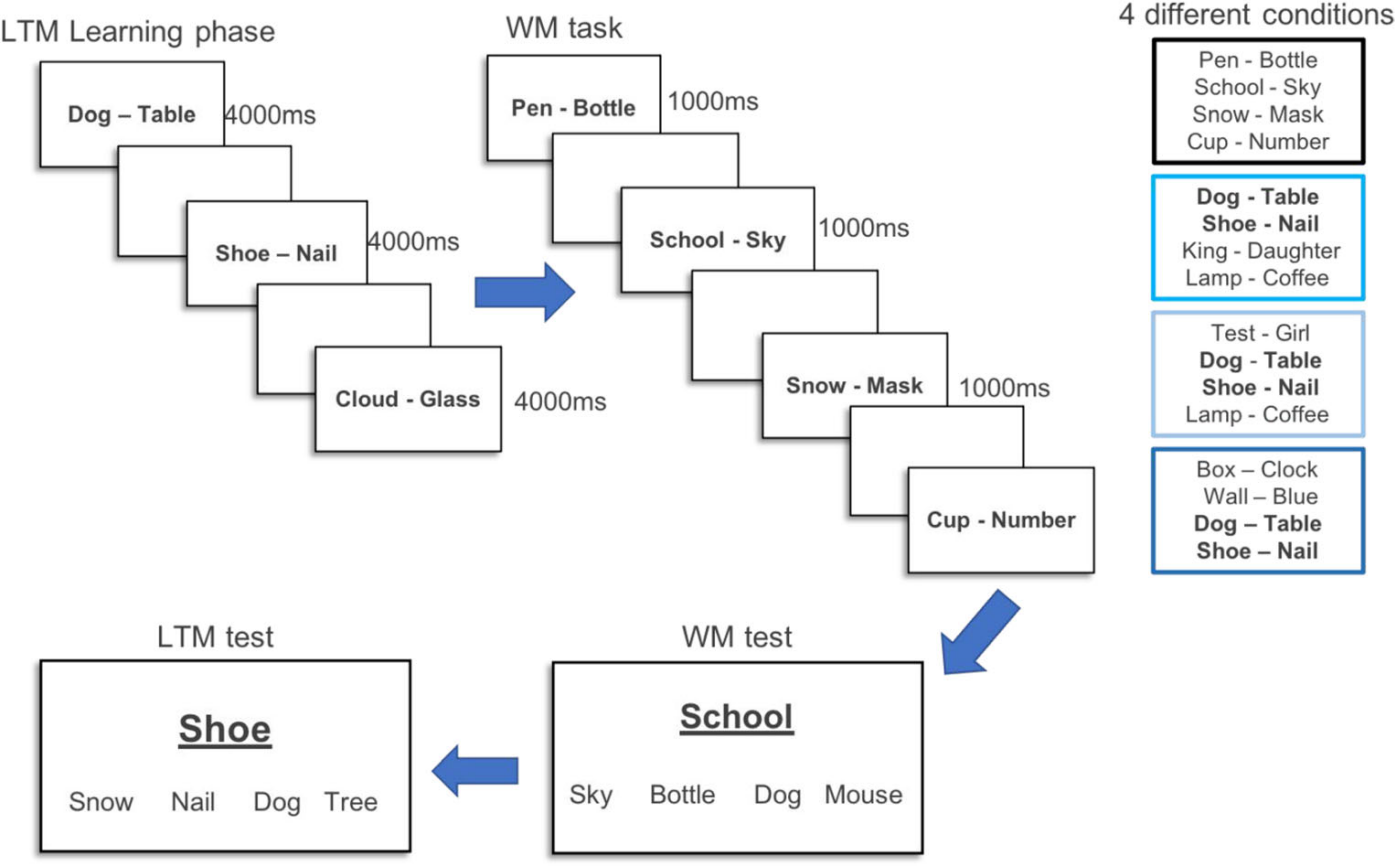
Study sequence of Experiment 1, with the long-term memory (LTM) learning phase, the working memory (WM) task and test, and the LTM test. Examples for the four different conditions (new pairs only, LTM first, LTM middle, and LTM last) are presented on the right

The experimental task was based on that used in a recent study (Bartsch & Shepherdson, 2022), and was adapted to investigate our specific research question. Figure 1 provides an overview of the general procedure of Experiment 1. The experiment consisted of three phases: an LTM learning phase in which subjects were simply presented with 42 pairs of concrete words, a subsequent WM task phase in which four word-pairs were presented sequentially, and a final LTM test of memory for the pairs presented in the previous two phases. Stimuli consisted of random pairs of 360 concrete English nouns. As in our previous study, the learning phase simply entailed the presentation of the material to the participant for 4,000 ms, with an inter-stimulus-interval (ISI) of 1,000 ms, with no test prior to the WM phase

In the WM task, participants viewed four pairs of words (e.g., cat-table) for 1,000 ms each, separated by a 500-ms ISI, and subsequently were tested on their immediate memory for the pairs in random order using a four-alternative forced choice task (4-AFC). In the 4AFC task, testing immediate memory (= WM Test), they had to use the mouse to select the correct associate (e.g., table) from four options when given its partner (e.g., cat) as a cue. The four response options consisted of the target (previously paired with the cue), another item that had been paired with a different word in the same trial (within-trial intrusion probe), an item that had been presented in the LTM learning phase but not the current WM trial (LTM lure), and a new item. In trials including LTM pairs, the within-trial lure randomly came from a within-trial LTM pair or a new pair. LTM was tested the same way, with response options including the target (previously paired with the cue), another item that had been paired with a different word in the WM task phase (WM lure), an item that had been presented in the LTM learning phase but not the current WM trial (LTM lure), and a new item.

By implementing such a relational memory task, we are able to ensure that the search set at test can specifically measure binding memory – rather than a familiarity signal for items – representing the core of WM functioning (for similar approaches and details, see Bartsch & Shepherdson, 2022, and Wilhelm et al., 2013).^2^ If participants do not have intact binding memory in WM, they would choose between the target and within-trial intrusion probe with equal probability. If their response was driven by long-term memory familiarity, they would more frequently choose the LTM lure. Finally, if subjects have no memory at all, then they would guess between all four options with equal probability.

The memory lists used in the WM phase consisted of either new pairs only – meaning that all word pairs were new – and lists in which two of the four pairs had been pre-learnt at the beginning of the experiment. This set-up allows us to investigate the benefit of the presence of LTM-available information within a WM list, by comparing the performance in these LTM-available trials (i.e., two new pairs + two LTM pairs) relative to trials with the same set size composed of only new pairs (e.g., four new pairs). Critically, here we controlled for the input position of the LTM pairs within a list: LTM pairs were presented at the beginning (serial positions 1 and 2), the middle (serial positions 2 and 3), or the end of the list (serial positions 3 and 4). This resulted in four conditions that were realized within-subjects: *new pairs only*, *LTM pairs first*, *LTM pairs middle*, and *LTM pairs last*.

If the benefit of the presence of LTM pairs is specifically driven by freed-up WM resources that can be used to encode subsequent new pairs (as per the chunking account), the presence of these LTM pairs *at the beginning* of a memory list should allow for a greater performance benefit compared to when they are presented *at the end*. By contrast, if participants benefit from the presence of LTM pairs because their greater trace strength facilitates retrieval from episodic memory, the position of LTM pairs should have no influence on performance.

The final part of the study consisted of an LTM test for the pre-learnt pairs (LTM pairs) used in the experiment. Here, participants were presented with the same 4-AFC procedure. Performance in this phase 3 LTM test was used as an index of successful learning.

Data were collected online via Prolific. The 20-min experiment comprised 28 WM trials in total, with four trials per condition that occurred in random order.

#### Data analysis

The dependent variable of interest was the proportion of correct responses in the 4-AFC test as a function of condition (*new pairs only, LTM pairs first, LTM pairs middle, LTM pairs last*) and serial position (1–4). Correct responses were defined as recalling the target item from the four alternatives (target, within-list lure, LTM lure, or new item).

We analysed the data using Bayesian mixed-effect models (LME) using the *lmBF* function implemented in the *BayesFactor* package (Morey et al., 2015) in R (R Core Team, 2017). With this we calculated Bayes factors (BFs), which represent the strength of evidence for a specified model (M1) against a Null or reduced model (M0). For instance, we can calculate the evidence for the effect of *condition* (BF_10_) by comparing the evidence for a model including this factor against an intercept-only model that serves as the null model. Additionally, we can calculate evidence against an effect of *condition* (BF_01_), where BF_01_ = 1/BF_10_. A BF_10_ larger than 1 gives evidence for an effect, a BF_10_ lower than 1 yields evidence against an effect and hence evidence for the null hypothesis. A BF_10_ of 10 indicates that the data are ten times more likely under the alternative hypothesis than under the null hypothesis. Usually, BFs below 3 are regarded as *anecdotal* evidence and BFs > 3 are regarded as providing *substantial* evidence for one hypothesis over the other (Wagenmakers et al., 2011). All models included a random intercept for participant, and random slopes over participants for the effects of the variables manipulated within subjects (Barr et al., 2013).

### Results

All data and analysis scripts can be accessed on the Open Science Framework (https://osf.io/49wa5).

#### Does LTM for word pairs enhance immediate memory performance?

First, we ensured that subjects had built-up LTM for the pre-learnt pairs. Results showed that subjects correctly responded to 64.6% (SD = 18.2) of the pairs in the final test (where chance performance in the 4-AFC task is 25% correct). We then turned to the data from the WM task: Performance was superior when the WM set included pre-learnt (i.e., LTM) word pairs than when it consisted entirely of new pairs (BF_10_ = 24.17).

#### Does LTM pair position influence *immediate memory* performance?

**Fig. 2:**
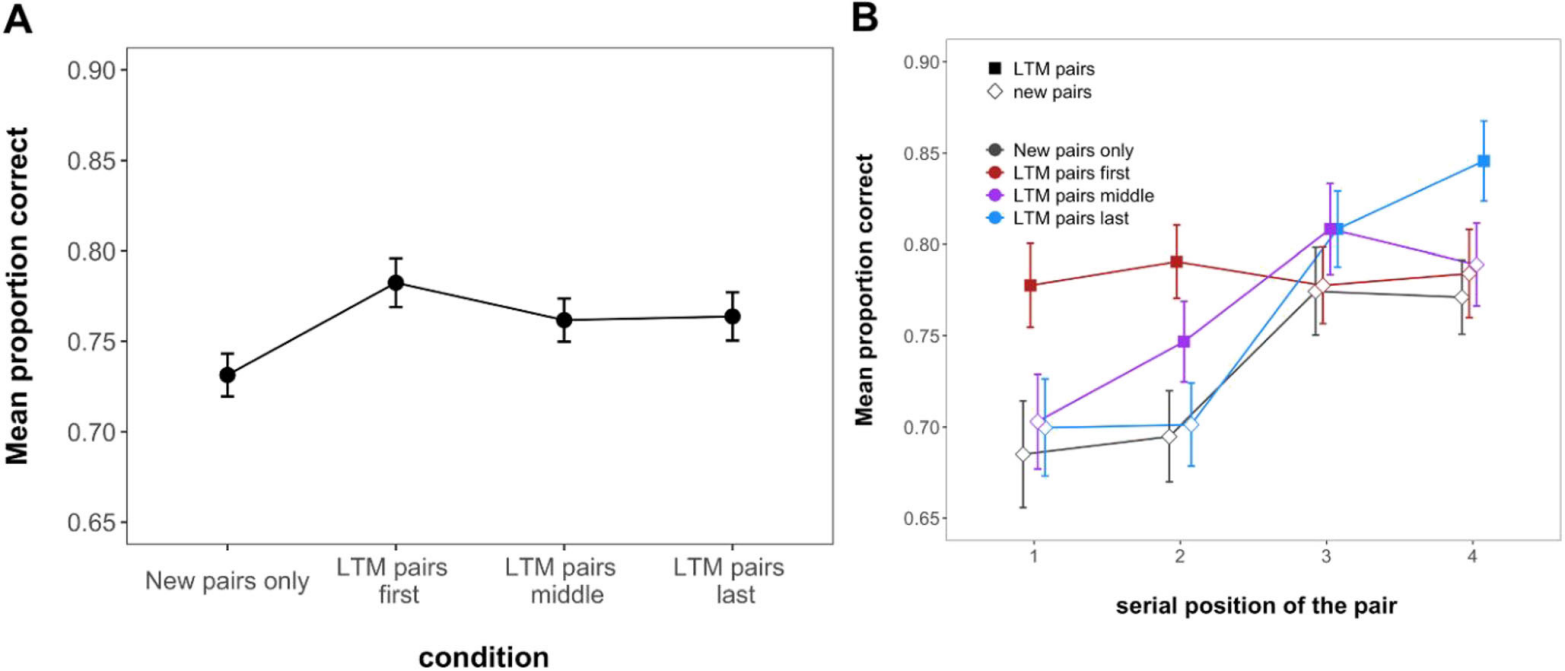
Mean immediate recall performance in Experiment 1. **a** shows performance across all conditions; **b** shows the performance in all conditions across serial positions. Error bars represent within-subject confidence intervals

As seen in Fig. 2a and supported by our analyses, the position within the WM set at which LTM pairs were presented did not influence overall task performance: Memory was superior in trials containing LTM pairs, irrespective of whether these pairs were presented in the initial two positions, the middle two positions, or the final two positions (main effect of condition BF_10_ = 7.18; for pairwise comparisons see Table 1). Contrary to our previous findings (Bartsch & Shepherdson, 2022, Experiments 1 and 2), despite a modest numerical advantage, memory for new pairs did not meaningfully differ between conditions containing LTM pairs (irrespective of their position) and the new pairs-only condition (BF_01_ = 5.22), indicating that the presence of LTM pairs did not provide an advantage to memory for new pairs.

**Table 1:** Bayes factors (BFs) of the pairwise comparisons of *the main effect* of condition on working memory (WM) performance of Experiment 1

	LTM *pairs* first	LTM *pairs* middle	LTM *pairs* Last
New pairs only	222^a^	3.06	2.12
LTM *pairs* first		0.28	0.28
LTM *pairs* middle			0.38

BFs > 3 represent substantial evidence for better performance in the conditions listed on the top compared to the ones listed on the left. BFs < 1/3 reflect substantial evidence against a difference between the conditions

^a^The interested reader can find posterior density plots of the comparisons in the Online Supplementary Materials (OSM)

Examining performance across serial positions, however, showed a more complex pattern. As shown in Fig. 2b, there was a credible interaction between condition and serial position (BF_10_ = 9.83 × 10^5^). Specifically, post hoc comparisons indicated that LTM pairs were remembered better than new pairs in the first two serial positions and the last (BF_10_ = 91.53, and BF_10_ = 78.17 BF_10_ = 18.00, respectively), irrespective of the condition. Further there was a general recency effect for all pairs, reflected in better performance at SP 3 and 4 versus 1 and 2 for the new pairs only, LTM pairs middle, and LTM pairs last conditions (BF_10_ = 7323, BF_10_ = 5839, BF_10_ = 1.61 × 10^11^, respectively). The latter may indicate that there is an additional performance boost of the information being retrieved from the focus of attention (FoA).

#### Contribution of the focus of attention

Following the unexpected finding of a general recency effect for all pairs in Experiment 1 – even though all pairs were tested in random order – we inspected the effect of input-output distance on the probability of choosing the correct response. The input-output distance represents the number of intervening events – encoding or testing of other pairs – between encoding of a given pair and the test of that pair. For example, if a pair is presented in the serial position 3, and tested first, the input-output distance would be 2. If the same pair were tested fourth, the input-output distance would be 5. This analysis was exploratory, as we had no predictions for it, but it turned out to be informative about the contribution of the FoA. Specifically, the additional performance boost of the information being retrieved from the FoA should only hold for the specific case in which the last presented pair is tested first (i.e., with an input-output distance of 1). In all other testing orders, the last presented pair would no longer be in the FoA as other pairs would have been tested and retrieved before it.^3^

**Fig. 3:**
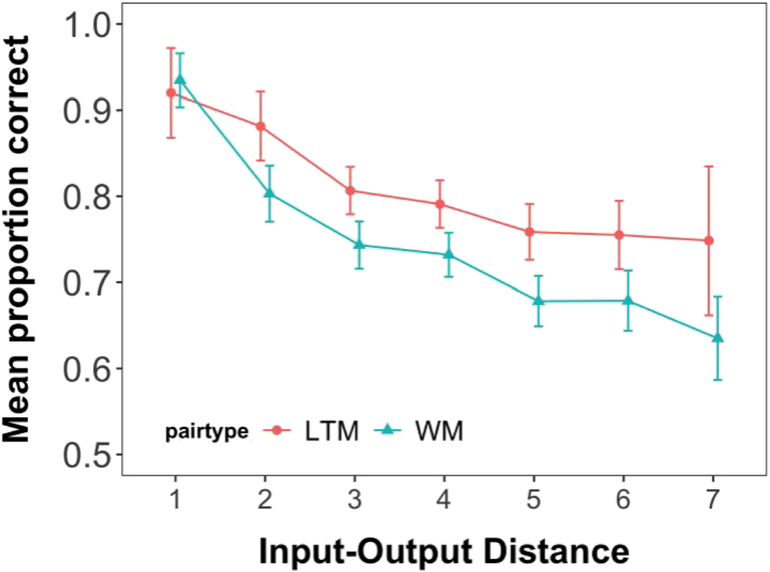
Probability of choosing the correct response option as a function of pair type and input output distance in Experiment 1. Error bars show the between-subjects confidence intervals

As seen in Fig. 3, the boost to performance when the last pair was tested first was about 15% for new pairs, compared to larger input-output distances. When considering that such trials only reflect one-quarter of all tests of the last-presented item, this corresponds to a last-item benefit of about 4.25%. In Fig. 2b, we can see that none of the conditions has last-position performance that is superior to penultimate-position performance by more than this value, suggesting the data are consistent with this account. Further, for LTM pairs, performance was likewise best for pairs that were presented last and tested first. Additionally, the effect extended to the second shortest input-output distance.

### Discussion

Experiment 1 showed superior performance when LTM pairs were included in the WM task. However, *where* the LTM pairs were included did not seem to matter. This pattern is inconsistent with the chunking account, but more consistent with the trace strength account: that having access to existing traces in LTM enhances episodic retrieval during the WM task.

However, there are also some reasons to doubt the effectiveness of this account in explaining our results. One such reason lies in the strong recency effect – independent of pair type – that was evident in the data. According to the logic of the trace strength account, the benefit of having information stored in LTM should be greatest in situations where the WM trace would otherwise be weakest, and least where that trace would otherwise be strongest. For instance, consider a situation in which a participant must choose between responses that are selected probabilistically based on their relative activation levels, according to the choice rule

$$ p(i)=\frac{A(i)}{\sum_{j=1}^nA(j)}, $$

(e.g., Luce, 1959), where *p(i)* is the probability of selecting response option *i*, *A(i)* is the memory activation of the item corresponding to that response, and *j*=1…*n* is the set of possible response options (including i) (e.g., Oberauer, & Lewandowsky, 2019). For illustration purposes, simplify the scenario to one where the choice is between only two responses: *C* (the correct response) and *L* (a lure). When the memory trace supporting *C* is at its weakest (i.e., non-existent), and *A(C)* → *A(L)*, the probability of selecting *C* and *L* is almost identical: *p(C)* = *p(L)* = .5. In this situation, adding a boost to *A(C)* from episodic LTM should have a pronounced effect on the probability of selecting the correct response. By contrast, when the memory trace supporting *C* is at its strongest, and *A(C)* ≫ *A(L)*, the additional advantage obtained from an extra boost from LTM will be minimal. Contrary to this, our data show an advantage for LTM over new pairs in the final list position (where performance in the condition with new pairs only was at or close to its best) that is similar in magnitude to the advantage at the second serial position (where performance in the condition with new pairs only was close to its worst).

It may also be premature to dismiss the chunking account – or something conceptually similar – entirely on the basis of these data. Our claim that this account would predict position-specific benefits of LTM availability was based on the premise that participants would need to encode the constituents of a pair separately before being able to recode it as a reference to a chunk already present in LTM, consistent with the account that best explained Thalmann et al.'s (2019) data. However, in addition to using pairs of words as our stimuli, rather than letter triplets, our procedure also differed from Thalmann et al.’s in the manner of presentation: Both words in a pair presented simultaneously, rather than constituent parts of a chunk presented consecutively. It could be argued that having both words present on screen reduced the need to encode the individual words into WM prior to chunking, or obviated the need to encode them at all if they were recognized as a pre-learnt pair. If so, this may have allowed LTM pairs to reduce the load on WM irrespective of the position in which they were presented. Unpublished data from Musfeld, Mizrak, and Oberauer, however, has shown that Thalmann et al.’s results – of a greater chunking benefit for chunks presented at the beginning of a WM trial – hold when the constituent parts of letter chunks are presented simultaneously, rather than sequentially. As such, though it is possible that participants in our experiment were able to offload the LTM pairs to LTM without first encoding them in WM, if so, this ability is unlikely to be a function of simultaneous rather than sequential presentation.

We are thus left with two imperfect accounts of our data: The trace strength account, and a revised chunking account (hence, an *offloading* account) in which pairs can be offloaded to LTM without prior encoding in WM. The predictions of the trace strength account seem inconsistent with the fairly large LTM advantage even when immediate memory performance in the WM task is good; and an account in which LTM pairs are offloaded and capacity in WM freed, which would likely predict that memory for new pairs also benefits from the offloading of LTM pairs, seems inconsistent with the limited difference between conditions when only new pairs were considered.

How might these accounts, imperfect as they are, be distinguished? One possibility lies in the effect of disruption on WM on immediate memory performance, in the form of interfering or distracting stimuli. Distractors have been shown in the past to interfere with more transitory forms of memory (e.g., representations the FoA and region of direct access of WM), but not with LTM (Bartsch & Oberauer, 2021; Oberauer et al., 2018).

According to the trace strength account, the advantage of having an existing trace in LTM is maximized when immediate memory would otherwise be poor. This is because the boost of activation provided by the LTM representation makes discriminating correct and lure responses easier under these circumstances but has a relatively limited effect when the discrimination is already easy. In the presence of interfering stimuli, we would thus expect immediate memory performance to generally become worse and the effects of prior LTM representations to become more pronounced. At the same time, despite the greater difference between immediate memory for stimuli with and without existing LTM representations, memory for those stimuli boosted by LTM should also become worse, because whatever contribution the non-LTM representation makes to performance for the LTM-available pairs will be decreased.

According to the offloading account, in which information is offloaded to LTM where possible without prior encoding to WM, we would also expect generally worse immediate memory performance, as the interfering stimuli compete with the existing contents of WM. Similarly, we would also expect an increase in the difference between LTM-available and novel word pairs. However, in this account this increased difference results from a selective influence of interfering stimuli on the novel pairs: The LTM-available pairs are offloaded to LTM, and should thus be immune to such interference.

As such, the two accounts differ in their predictions about how selective the effects of interfering stimuli would be on LTM-available versus unavailable pairs. We addressed this issue in our second experiment.

## Experiment 2

In Experiment 2, as in Experiment 1, participants first learnt 16 arbitrary word pairs. Then, they completed the WM phase of the experiment, in which they were presented with sets of four word pairs – consisting of the same mixtures of LTM-available and new pairs as in Experiment 1 – to remember. We also used the same immediate memory testing procedure, with participants selecting the correct word from amongst lures, given its partner as a cue. However, here we used an extended (10-s) retention interval, which was either blank or required participants to verify a series of simple equations.

As outlined previously, the trace strength and offloading accounts make similar predictions regarding the overall effect of the distractor-filled retention interval on immediate memory performance in the WM task, and on the difference between performance for LTM-available and LTM-unavailable pairs. However, whereas the trace strength account predicts inferior performance for both pair types, the offloading account predicts a specific deficit for LTM-unavailable, new pairs.

Experiment 2 was also designed to evaluate the role played in performance by the focus of attention (FoA). None of the accounts we have discussed explicitly predicts the superior performance in the final list position(s) that was evident in all conditions in Experiment 1. By simultaneously evaluating the effect of an expanded retention interval, and the presence of distractors within that interval, we hoped to shed light on the way the FoA contributes to performance in the task, and on its interaction with memory for both types of pairs. We had no specific predictions concerning the form this interaction would take, but generally expected the performance advantage for later serial positions to be minimized in the condition with distractors presented during the retention interval.

### Method

#### Participants

We collected data from 150 new participants online via Prolific (M_age_ = 25.44 years, 107 female). We made sure that none of the participants who participated in Experiment 1 took part in Experiment 2. We excluded 21 participants as their response accuracy in the final LTM test was below or at chance level. We further excluded six participants whose immediate memory performance was 2 standard deviations (SDs) below the mean. Therefore, the final sample included 123 participants. All participants’ first language was English.

#### Materials and procedure

**Fig. 4:**
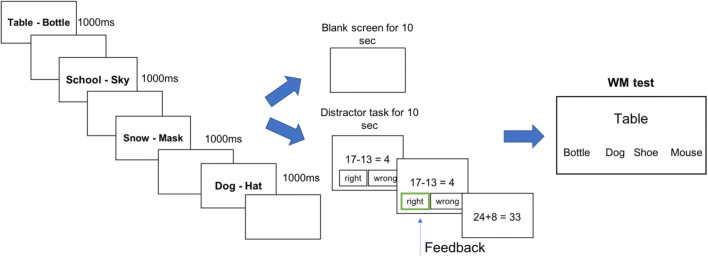
Working memory (WM) task of Experiment 2. List presentation (according to the four conditions: new pairs only, long-term memory (LTM) first, LTM middle, LTM last), is followed by a distractor task in half of the trials

Apart from the changes detailed in the following, Experiment 2 was consistent with the *Materials and procedure* used in Experiment 1. Here, half of the WM trials were followed by a blank retention interval of 10 s, the other half of the trials were followed by a 10-s distractor task. This distractor task entailed the judgement of the correctness of equations (e.g., 17−13 = 4?) until the time was up. The participants received feedback on their distractor task performance (see Fig. 4).

The 25-min experiment comprised 28 WM trials in total, with four trials per condition followed by a blank screen and three trials followed by the distractor task. The trials of different conditions occurred in random order and participants were unaware at encoding whether the retention interval would be blank or filled with the distractor task.

#### Data analysis

Apart from the changes detailed in the following, Experiment 2 was consistent with the analyses applied in Experiment 1. The dependent variable of interest was the proportion of correct responses in the 4-AFC test as a function of condition (new pairs only, LTM first, LTM middle, LTM last), serial position (1–4) as well as distractor (blank screen vs. distractor).

### Results

#### Does LTM for word pairs enhance immediate memory performance?

Again, we first ensured that subjects had built-up LTM for the pre-learnt pairs. Results showed that subjects correctly responded to 70.7% (SD = 18.5) of pairs in the final test (where chance performance on the 4-AFC is 25% correct). We also ensured that they engaged sufficiently in the distractor task. Results showed that subjects correctly responded to 93% (SD = 13.59) of the equations (where chance performance is 50 % correct).

**Fig. 5:**
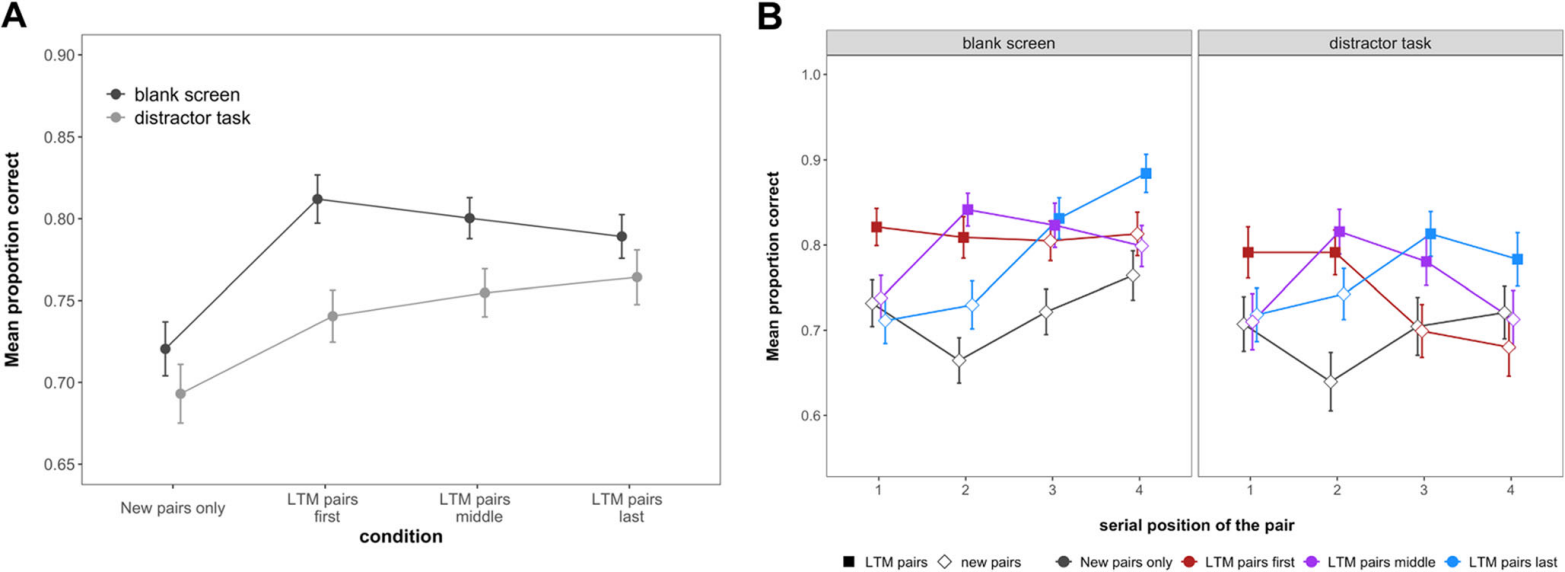
Mean immediate recall performance in Experiment 2. Panel (**a**) shows the performance across distractor task and condition. Panel (**b**) shows the performance for the conditions separately across all serial positions. Error bars represent within-subject confidence intervals

Replicating Experiment 1 and previous findings, the results of the WM task showed that immediate memory performance was superior when the WM set included pre-learnt (i.e., LTM available) word pairs than when it consisted entirely of new pairs (BF_10_ = 1.33 × 10^12^, see also Fig. 5a).

#### How does the inclusion of a distractor task in the retention interval influence the benefit obtained from LTM pairs?

As shown in Fig. 5a, whether or not a distractor task was included in the retention interval, immediate memory performance was superior when the memory set included LTM pairs than when it was composed exclusively of new pairs (main effect of condition: BF_10_ = 8.12 × 10^8^; all BF_10_ for paired comparisons between individual LTM-pair-containing conditions and the new pairs-only condition > 9.9 × 10^4^; all BF_10_ for comparisons across LTM-pair-containing conditions < 0.073). There was weak evidence that the inferiority of performance in the condition containing only new pairs was reduced when the distractor task was included (interaction: BF_10_ = 1.93). However, LTM-containing conditions showed superior performance to the new pair-only condition both with and without the distractor task (main effect of condition, with distractor task: BF_10_ = 111; without distractor task: BF_10_ = 2.94 × 10^7^). Further, and divergent from Experiment 1, when no distractors were presented during the retention interval, memory for new pairs was better in conditions containing LTM pairs than in the new pairs-only condition (BF_10_ = 3.84). This indicates that the presence of LTM pairs did free up WM capacity, which was then used to enhance memory for the new pairs.

Unsurprisingly, performance was also generally superior in the absence of a distractor task than in its presence (main effect of distractor: BF_10_ = 5.69 × 10^6^).

#### How does the inclusion of a distractor task affect recency effects?

In Experiment 1, recency effects were a feature of the data. Specifically, in all conditions – except the LTM pairs-first condition – memory for SP 3 and 4 was superior to 1 and 2. To assess whether the inclusion of the distractor task affected this pattern, we conducted a Condition × Distractor × Serial Position analysis. The data are shown in Fig. 5b. As anticipated, this showed a dramatic reduction in recency effects in the LTM-present conditions in the presence of a distractor task. Specifically, differences between distractor versus blank screen across the LTM conditions was only present in SP 4 (BF_10_ = 2.27 × 10^8^), but none of the others (SP 1: BF_10_ = 0.002, SP 2 BF_10_ = 8.8 × 10^-6^, SP 3 BF_10_ = 0.17). To assess the effect of distractors on memory for pre-learnt and new pairs across the LTM-available conditions, we investigated the effect for the different pair types separately: This showed that the LTM pairs were only affected by the distractor at SP 4 (BF_10_ = 72.17), but not at any earlier positions (SP 1: BF_10_ = 0.12, SP 2 BF_10_ = 3.39 × 10^-5^, SP 3 BF_10_ = 0.02), whereas the new pairs were also affected at SP 3 (BF_10_ = 44.18) and at SP 4 (BF_10_ = 4.39 × 10^5^). However, the new pairs were not affected by the distractor task at SPs 1 or 2 (BF_10_ = 0.01, and BF_10_ = 0.07, respectively).

Interestingly, the distractor task did not affect performance in the *new pairs-only* condition (BF_01_ = 3.58).

In the absence of a distractor task, we replicated the general data pattern from Experiment 1: credible interaction between condition and serial position (BF_10_ = 8.98 × 10^11^). Specifically, post-hoc comparisons showed that LTM pairs were remembered better than new pairs in the first two serial positions and the last (BF_10_ = 26.27, and BF_10_ = 5.48 × 10^9^ BF_10_ = 4405, respectively), irrespective of the condition. Further, there was a recency effect for all pairs, reflected in better performance in SP 3 and 4 vs. 1 and 2 for the LTM pairs last condition (BF_10_ = 7.53 × 10^9^). In contrast to experiment 1, the latter was not the case for the new pairs-only condition (BF_10_ = 0.02), nor the LTM pairs middle condition (BF_10_ = 5.83 × 10^-4^).

**Fig. 6:**
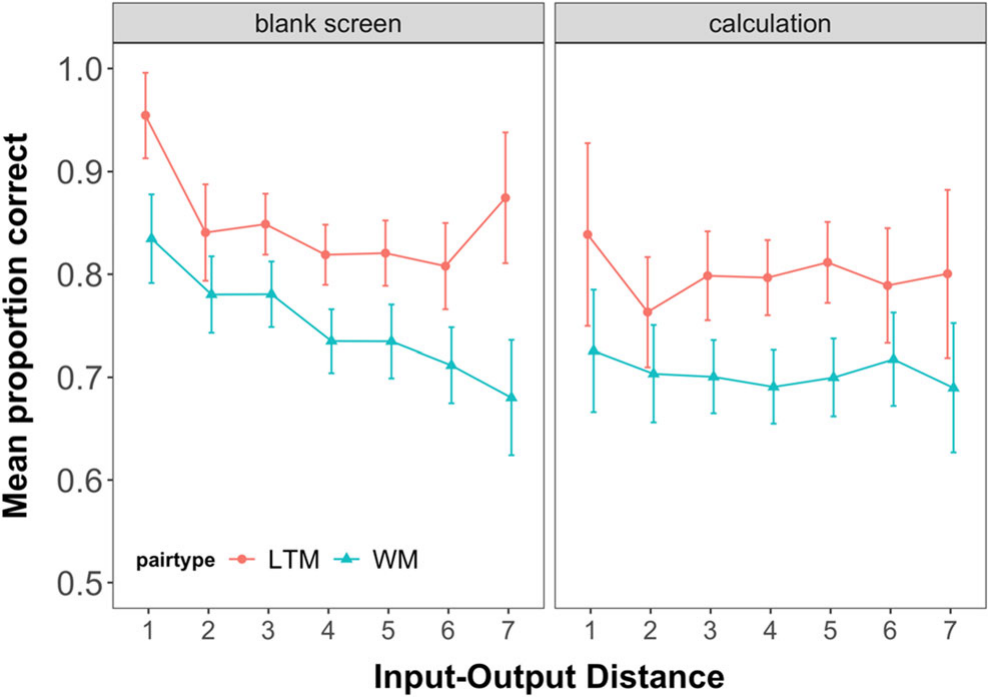
Probability of choosing the correct response option as a function of pair type and Input output distance in Experiment 1. Error bars show the between-subjects confidence intervals

As for Experiment 1, we also inspected the effect of input-output distance on the probability of choosing the correct response for Experiment 2. As seen in Fig. 6, the blank interval already reduces the benefit of the last pair being tested first drastically for the WM pairs. This is in line with previous work showing that in case a retention interval is inserted between the presentation of the list items and the probe, the last-presented benefit is no longer observed (Vergauwe & Langerock, 2017).

### Discussion

In Experiment 2, we aimed to discriminate between two accounts of the impact of episodic LTM on immediate memory performance: (1) a trace strength account, where representations stored in episodic LTM provide a boost to immediate memory performance in a WM task, and (2) an offloading account, where pairs with existing representations in LTM are not encoded in WM, but instead are “offloaded” to episodic memory. By comparing a condition incorporating a distractor task into the retention interval to one without, we hoped to determine whether interference selectively affects memory for new pairs (consistent with the offloading account) or affects both new and LTM pairs to different extents (consistent with the trace strength account).

Generally, our data were most consistent with the offloading account: Performance for new pairs was worse when distractors were presented during the retention interval than when the interval was blank, whereas performance for LTM pairs was largely unaffected.^4^ There were, however, some notable exceptions to this general pattern.

First, immediate memory performance for both new and LTM pairs was worse in the final serial position following a distractor-filled retention interval. This suggests that the distractors interfered dramatically with information held in the focus of attention. It appears that, in case of no distraction (in the blank-screen condition and in Experiment 1) the last item receives an additional boost from being held and retrieved from the focus of attention and in this state of heightened accessibility (e.g., Basak & Verhaeghen, 2011; Hitch et al., 2020; Nee & Jonides, 2008; Oberauer, 2002), which is no longer the case when a distractor task intervenes prior to test. Whereas prior research has shown such effects on response times, here, we found this last item benefit on response accuracy, despite the pairs being tested in random order.

Second, in the condition in which WM was loaded with four new pairs (i.e., in the new pairs-only condition), distraction had no effect on any of the serial positions. On the surface, this seems bizarre, as it suggests that in this condition, the last item does not receive the same boost from the focus of attention as occurs in the other three conditions. One possible explanation for this could be that retaining four new pairs (i.e., eight words in total) exceeded participants’ WM capacity, leading them to switch their attention away from the last-presented items in order to engage in additional strategies during the retention interval so that they could effectively recruit their LTM (e.g., elaboration; Bartsch et al., 2022). Another explanation draws on recent evidence showing that immediate memory performance in these types of binding tasks is driven by LTM at set sizes larger than three (Bartsch & Oberauer, 2021). With this, WM functions such that the focus of attention would have minimal impact on immediate memory performance at set size 4. We return to this possibility in depth in the *General discussion*.

Third, though the distractor task interfered with memory for new pairs at serial position 3 (in addition to the effect on both pair types at the final position), to our surprise the effects at positions 1 and 2 were negligible. Based on our conceptualisation of both the offloading and boosting accounts, we had expected the distractor task to have a negative impact on performance for new pairs at all serial positions, with the key difference between the accounts lying in whether the pre-learnt pairs would also be affected. If we accept the logic that distractors will interfere with more transitory forms of memory (e.g., the FoA and region of direct access of WM) but not with LTM, the conclusion we must draw from this is that LTM, rather than WM, performs the lion’s share of the work in remembering information at these earlier serial positions, and that direct contributions from WM are only relevant at positions 3 (with limited input from the FoA) and 4 (where the FoA also makes a large contribution).

Taken together, in Experiment 2 we have provided evidence that the benefit of including LTM pairs within a list of new pairs is best explained by an “offloading” account, but may also partly arise from a benefit of the last pair being in the focus of attention. This benefit vanishes, (1) in case a distractor task is included in the retention interval, and (2) if WM capacity is exceeded and performance is more heavily influenced by LTM in general. We revisit these ideas, focusing on consistency with prior results, in the *General discussion*.

## General discussion

In the present study we investigated whether the position of stimuli available in LTM within a list of new stimuli affects their impact on immediate memory performance in a WM task. Prior research evaluating the effect of semantic LTM on WM performance in a similar context had shown a position-dependent benefit resulting from the encoding and subsequent chunking of familiar stimulus configurations (Thalmann, 2019), leading us to hypothesize a similar process might arise when participants were presented with pairs of stimuli they had previously been exposed to. However, Experiment 1 showed a benefit from these LTM-available stimuli that was similar in magnitude irrespective of list position, more consistent with the idea that LTM availability provided a boost in activation that facilitated retrieval from episodic memory. In Experiment 2, we contrasted this account with an “offloading” account, in which LTM-available stimuli are not encoded into WM at all, freeing capacity for the retention of LTM-unavailable stimuli. In general, the results of this experiment were more consistent with the latter, with a distraction-filled retention interval resulting in inferior performance, predominantly for those pairs that lacked existing LTM representations.

In the following, we address three key questions that our results bear upon. First, in what ways do the contributions of semantic and episodic LTM to WM performance differ, and in what ways do they seem similar? Second, what role does the focus of attention play in performance on tasks such as those we used here? And third, what can our results tell us about theories of WM more broadly?

### Episodic and semantic contributions to WM: Similarities and differences

As outlined in the *Introduction*, a key motivation for these experiments was the work of Thalmann et al. (2019), whose chunking account of the benefits resulting from familiar stimulus triplets presented within a WM task provided our starting point to explain benefits of pre-learnt word pairs in our experiments. On the surface, the different patterns of results we found here – position-independent superior performance in conditions containing LTM-available stimuli, versus position-dependent benefits of familiar chunks in their experiments – could motivate speculation that different mechanisms may underly contributions to WM from semantic and episodic LTM.

However, an alternative to this view is the possibility that differences across our experiments and theirs result from differences in the stimuli participants needed to remember, and the methods used to test memory. First, whereas Thalmann et al.’s (2019) participants were mostly required to recall letters in forward serial order, in our experiments participants were given one word in a pair as an explicit cue to select the other from a small set of choice options. These two procedures make different demands on memory. For instance, in serial recall, the binding of items to positions is vital; whereas in our procedure such positional information would help performance only tangentially (e.g., if a participant remembers which individual words were presented in which positions and uses that positional information to infer the appropriate pairs). Serial recall requires the retention of information about the relative positions of stimuli, implying that even if some of those stimuli can be chunked, the position of the chunk relative to any non-chunked stimuli must still be retained. By contrast, upon identifying a familiar pair in our experiments, the position at which that pair was presented became irrelevant: As long as a participant remembered the pairing itself, this information was sufficient to produce a correct response when tested. This may have allowed participants to avoid encoding pre-learnt pairs in WM entirely, in a way that was not practical for Thalmann et al.’s participants: Because the LTM representation of the word pair contains the necessary binding information, no additional binding of the pair to a context is necessary.

Second, all the pre-learnt pairs participants saw in our experiments were reproduced exactly in the WM trials. For example, if a pair in the learning phase had been *shoe-pen*, then *shoe* was only ever presented in the WM phase when paired with *pen*. That is, pairs were never rearranged from one phase to the next (e.g., *shoe-pen* in the learning phase, then *shoe-apple* or *apple-pen* in the WM phase). By contrast, the familiar three-letter chunks that Thalmann et al. (2019) presented to their participants were not the only context in which the letters comprising those chunks were displayed. For example, the familiar initialism *F-B-I* could not have been perfectly anticipated upon presentation of its first two letters, because the less meaningful *F-B-Q* was also a potential stimulus. This difference in stimulus entropy across experiments could be responsible for the different patterns of results: A context where a stimulus may not be easily “chunkable” upon presentation could incentivize initial encoding of the chunk’s constituent parts, preventing the apparently complete offloading to LTM that appears to have occurred in our study. This possibility is supported by the fact that, in our prior research (Bartsch & Shepherdson, 2022), the advantage obtained for memory for novel pairs through the presence of pre-learnt pairs was minimized when the words within those pre-learnt pairs could be rearranged, inducing proactive interference in the WM phase. In other words, it seems that offloading of this sort may be disrupted in situations where the material stored in LTM is an unreliable guide to WM task performance.

To properly clarify which aspects of the differences across tasks result from distinctions between contributions from episodic and semantic memory, and which reflect the influence of different methodological features will require deliberate manipulation of these features. For instance, a future experiment in which semantically related word pairs (e.g., *coffin-grave*) are used in place of arbitrary pre-learnt pairs may help to evaluate the importance of the episodic/semantic distinction. Alternatively, building on Thalmann et al.’s (2019) first experiment, pre-learning of arbitrary word pairs that are then incorporated into a WM set and must be recalled in serial order may help to isolate the importance of serial recall in positional effects of LTM stimuli on WM performance.

What *is* clear from both sets of experiments, in addition to the broader body of literature evaluating the effects of LTM on WM performance, is that there appear to be multiple paths by which such information can exert an influence. Whether this takes the form of easier stimulus encoding for familiar materials (e.g., Xie & Zhang, 2017, 2018), the ability to store information more compactly (e.g., Thalmann et al., 2019), or the offloading of information to free capacity (e.g., Bartsch & Shepherdson, 2022), the knowledge we have seems to benefit us in numerous ways.

### How does the focus of attention contribute to performance?

In Experiment 1, and in the blank-screen condition in Experiment 2, WM performance was superior in later serial positions – particularly the last – than in earlier positions, across all conditions. This led us to posit a contribution to last-position performance from the focus of attention (FoA), and provided additional motivation for our inclusion of a condition in Experiment 2 in which participants performed a distractor task during the retention interval. Perhaps unsurprisingly, the condition with the distractor task showed inferior performance (relative to the blank-screen condition) almost exclusively at position 4, which we take as indicating that whatever enhancement the FoA had offered was no longer available. However, this finding was complicated by the fact that the difference across conditions seemed entirely absent when the memory set consisted solely of new pairs. If anything, our (admittedly tentative) expectation prior to data collection had been that performance in this condition would be *most* affected by the distractor task, being a condition in which WM capacity was most heavily taxed and therefore the FoA could be expected to contribute most substantially. How can we explain this apparent contradiction?

Earlier, we suggested that the sheer amount of novel information needing to be retained in the condition containing only new pairs may have been responsible. According to this line of reasoning, WM is so taxed in this condition that participants must engage additional strategies – such as elaboration or refreshing – to effectively remember the material (Camos et al., 2009; Loaiza et al., 2011; but see Bartsch et al., 2018). Assuming the use of such strategies requires the FoA, then whatever benefit would otherwise be derived from simply holding an item in the FoA would likely be diminished, and the enhanced memory for the final item(s) that it would otherwise provide would not be present in this condition. Thus, occupying the FoA with a distractor task has a smaller effect on final-position memory relative to other conditions.

Admittedly, this account has some rather awkward implications, although it could explain the relatively minimal difference between the distractor and blank-screen conditions at position 4. The awkward implications are as follows: If the FoA is required for whatever memory strategies participants engage, it is not clear why the inclusion of a distractor task would not produce substantially worse performance at *all* positions when all memory stimuli are novel. That is, if the FoA is used to engage in strategies that facilitate memory in this condition, then all else held equal we would expect that occupying the FoA during the retention interval would lead to worse performance across the board. This is clearly not borne out in the data, where differences between the blank-screen and distractor-task conditions were minimal (i.e., *BF* = 3.57 in favour of the null) in trials in which only new pairs were presented.

An alternative explanation is based on the premise that, rather paradoxically, the condition containing only novel pairs was that in which participants relied most heavily on episodic LTM. Evidence for this claim comes from a recent study investigating the contribution of episodic LTM in a WM binding task, similar to the one we used here: Bartsch and Oberauer (2021) varied set size of to-be-remembered word-picture pairs and showed that immediate memory performance at set sizes larger than 3 was specifically affected by proactive interference – but were immune to influences from a distractor-filled delay. In contrast, performance at set size 2 was unaffected by proactive interference but harmed by a distractor-filled delay. According to this account, because of the load on WM when four novel pairs must be remembered in the present study, and the difficulty in retaining that much information, participants are more inclined to rely largely or exclusively on a one-shot episodic LTM rather than attempting to store the relevant information in immediate memory or the FoA. By contrast, in conditions with pre-learnt pairs, the offloading of this information means sufficient capacity is available in immediate memory to be allocated to the novel pairs, and the FoA contributes by providing an extra boost to memory for whatever information it happens to contain – most frequently whatever was presented last. Thus, using the FoA to solve distracting problems negatively affects memory for the final position in LTM-available trials, but has only a minimal effect in trials consisting entirely of novel pairs.

This account predicts that physiological markers of WM load, such as the contralateral delay activity (CDA; e.g., Rajsic et al., 2019; Vogel & Machizawa, 2004), should be greater in LTM-available conditions than in LTM-unavailable conditions. We are currently working on evaluating this possibility, in comparison to an account in which offloading of information to LTM results in the opposite prediction (i.e., a smaller CDA in LTM-available conditions).

### Implications for theories of WM

In our previous work (Bartsch & Shepherdson, 2022), we drew on accounts of WM that characterize it as a system that relies on and interacts with LTM (e.g., Beukers et al., 2021; Cowan, 1988, Cowan, 1995; Oberauer, 2002; Unsworth & Engle, 2007a, 2007b), binding the contents of LTM to novel contexts in response to task demands (e.g., Oberauer, 2019). We also emphasized that the benefit of LTM to performance in WM tasks depends on the utility provided by pre-learnt information (e.g., its reliability as a guide to what someone should do), and the ease with which decisions about whether to offload information to LTM (i.e., not to expend resources creating bindings to novel contexts) can be made. How can our present results be interpreted in light of these ideas?

First, though we previously found that being able to rely on information in LTM produced consistent benefits when assessing memory for novel information (Bartsch & Shepherdson, 2022), the results from our two experiments here were mixed in this regard. Specifically, whereas we found no advantage for new pair memory in LTM-available conditions in Experiment 1, in Experiment 2 the benefit we had demonstrated previously was present when the retention interval was blank, but absent when it was filled with a distractor task. The latter – no benefit with distractors, but a benefit in their absence – is consistent with the idea that any WM capacity freed through offloading to LTM was subsequently occupied in processing the distractors, negating this advantage. However, the lack of a benefit in Experiment 1 is surprising, given that this experiment was very similar to those that, in our previous work, had shown such an effect. Based the totality of the results, we believe that the most parsimonious explanation for this inconsistency is simply noise in the data. Nonetheless, it may be worthwhile to more systematically investigate various potential procedural explanations for this discrepancy in future.

Second, the fact that the deterioration of memory for pairs presented in the final serial position (i.e., position 4) that occurred following a distractor task in Experiment 2 was present for both novel and pre-learnt pairs suggests that people do not simply rely exclusively on LTM even when LTM is a reliable guide to what action they should take (here, what response they should select). Rather, it indicates that different sources of memory – such as LTM and the FoA – make contributions to performance to the extent that people judge the information they provide to be useful. Thus, when a distractor task occupied the FoA, this led to a reduction in response accuracy irrespective of whether the word pair being tested was pre-learnt or novel.

By contrast to data from the final position, when evaluating response accuracy in the penultimate serial position in the same experiment, the distractor task seems to have had a much more pronounced detrimental effect on novel pairs than on those that were pre-learnt. Specifically, comparing performance between the LTM pairs first and LTM pairs middle conditions at serial position 3 shows similar performance in the absence of a distractor task, but relatively superior performance in the latter condition (in which the third pair was pre-learnt) when the distractor task was present. This can be reconciled with the similar effects of distractors on novel and pre-learnt pairs in position 4 by assuming that (a) the distractor task disrupted both the FoA and region of direct access of WM; (b) the contribution of the FoA to performance was similar across novel and pre-learnt pairs; (c) the contribution of WM to performance was greater for novel pairs than for pre-learnt pairs; and (d) in the absence of distractors, the FoA typically retained information about the last-presented pair (see Oberauer & Hein, 2012, for a review). In short, the distractor task disrupted both types of pairs in the final serial position because the FoA was used to support performance irrespective of pair type, whereas the disruption was greater for novel pairs at position 3 because the region of direct access of WM was used for novel pairs, with pre-learnt pairs offloaded to LTM. Yet this remains speculation and needs further testing.

An implication of this line of argument is that the contribution of WM to performance at positions 1 and 2 was fairly limited irrespective of pair type (because the effect of the distractor task was smaller at these positions). Though at first this may seem odd, if performance in an immediate memory task is conceptualized as reflecting contributions from both WM and episodic memory (e.g., Bartsch & Oberauer, 2021; Beukers et al., 2021; Raaijmakers & Shiffrin, 1980; Shiffrin & Atkinson, 1969; Unsworth & Engle, 2006a, 2006b, 2007a, 2007b) , it can be explained by positing that memory for the material presented in these early positions was largely the responsibility of episodic LTM. In future work, we hope to build on the idea that different contributions from different potential memory sources (e.g., the FoA and region of direct access in WM, and LTM) lead to distinctive patterns of performance in the type of task we have used here and previously (Bartsch & Shepherdson, 2022), by creating and contrasting the predictions of different computational models of the task.

## Conclusion

The experiments reported here build on a body of research showing that LTM can facilitate immediate memory performance in a WM task in a multitude of ways. In particular, a comparison of past work and our present findings suggests that LTM is particularly beneficial when the representations we rely on include bindings that would otherwise need to be generated from scratch. In sum, our data support an account of WM in which different sources of information are flexibly combined to optimize task performance. The serial position at which prior knowledge in the form of pre-learnt stimuli is presented did not affect this.

## Supplementary information

ESM 1 (DOCX 1096 kb)

## Footnotes

1 We refer to tasks that require the immediate retrieval from memory as immediate memory tasks. These are commonly used to assess the contents of WM.

2 Specifically, Wilhelm et al. (2013) provided evidence that a general binding factor derived from this type of task represents a common source of variance in typical WM tasks (e.g., complex span, Updating, Recall-1-back; Wilhelm et al., 2013), which is why we chose to implement this paradigm here as well.

3 We thank an anonymous reviewer for pointing us to this aspect of the data.

4 One insightful reviewer suggested that a sufficiently large “boost” from LTM could overcome distractor effects, leading to a situation where LTM pairs are essentially resistant to distractors. We implemented this idea as a simple formal model and found the reviewer’s suggestion to be correct. However, the model was not able to simultaneously account for this and other relevant features of the data (e.g., it required a larger advantage for LTM vs. new pair performance than is evident empirically), leading us to consider the strengthening account generally inferior in comparison to the offloading account – though by no means totally implausible.
