# Supplementary material for: Chunking, boosting, or offloading? Using serial position to investigate long-term memory's enhancement of verbal working memory performance
Source: Atten Percept Psychophys. 2022 Dec 1;85(5):1566–81. doi: 10.3758/s13414-022-02625-w (PMC10371913; doi:10.3758/s13414-022-02625-w)

**Supplementary Material**

**Figure S 1**

Posterior densities of the pairwise comparisons of the LTM conditions to the new pairs only condition. Points show the posterior modes and the lines the 95% (thick lines), and 99% (thin lines) highest-density interval.


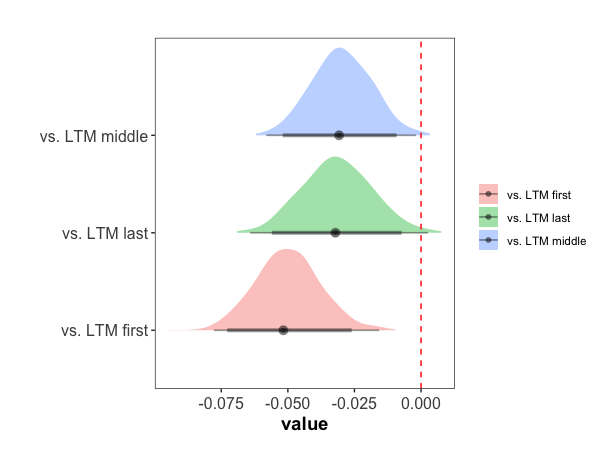

Supplement: Supplementary file 1 — (DOCX 1096 kb) [file 13414_2022_2625_MOESM1_ESM.docx]
